# Mechanical Stimulation by Postnasal Drip Evokes Cough

**DOI:** 10.1371/journal.pone.0141823

**Published:** 2015-11-18

**Authors:** Toshiyuki Iwata, Isao Ito, Akio Niimi, Koji Ikegami, Satoshi Marumo, Naoya Tanabe, Hitoshi Nakaji, Yoshihiro Kanemitsu, Hisako Matsumoto, Junzo Kamei, Mitsutoshi Setou, Michiaki Mishima

**Affiliations:** 1 Department of Respiratory Medicine, Graduate School of Medicine, Kyoto University, Kyoto, Japan; 2 Department of Medical Oncology and Immunology, Nagoya City University Graduate School of Medical Sciences, Nagoya, Japan; 3 Department of Cell Biology and Anatomy, Hamamatsu University School of Medicine, Hamamatsu, Japan; 4 Department of Pathophysiology and Therapeutics, Hoshi University School of Pharmacy and Pharmaceutical Sciences, Tokyo, Japan; University of Pittsburgh, UNITED STATES

## Abstract

Cough affects all individuals at different times, and its economic burden is substantial. Despite these widespread adverse effects, cough research relies on animal models, which hampers our understanding of the fundamental cause of cough. Postnasal drip is speculated to be one of the most frequent causes of chronic cough; however, this is a matter of debate. Here we show that mechanical stimuli by postnasal drip cause chronic cough. We distinguished human cough from sneezes and expiration reflexes by airflow patterns. Cough and sneeze exhibited one-peak and two-peak patterns, respectively, in expiratory airflow, which were also confirmed by animal models of cough and sneeze. Transgenic mice with ciliary dyskinesia coughed substantially and showed postnasal drip in the pharynx; furthermore, their cough was completely inhibited by nasal airway blockade of postnasal drip. We successfully reproduced cough observed in these mice by injecting artificial postnasal drip in wild-type mice. These results demonstrated that mechanical stimulation by postnasal drip evoked cough. The findings of our study can therefore be used to develop new antitussive drugs that prevent the root cause of cough.

## Introduction

Cough is a protective reflex that eliminates foreign materials and sputum from the airway. This important reflex prevents aspiration of foreign material and cleans the lower airway in cooperation with ciliary movement. Absence of the normal cough reflex increases the risk of developing pneumonia. However, many individuals are prone to excessive and intolerable cough, and acute and chronic cough impairs quality of life [1, 2]. Moreover, uncontrolled cough often causes adverse events such as syncope, urinary incontinence, bone fracture, muscle ache, and sleep disturbance.

The most frequent reason for seeking medical help is cough [3], and the economic burden of cough in the United Kingdom is estimated to be at least £104 million [4]. Various pulmonary and extrapulmonary diseases cause cough, and improvement of the causal disease is necessary for control. Antitussive agents are available for prompt inhibition of cough, even when the causal disease is not treatable. Unfortunately, there is insufficient evidence regarding the efficacy of antitussive agents used for the treatment of cough [5]. Therefore, there is an unmet need for more effective antitussive agents.

Rhinosinusitis accompanied by postnasal drip is regarded by many as one of the main causes of chronic cough [3, 4, 6], the association between postnasal drip and chronic cough remains controversial [6]. Recently, Kunimoto et al. and we observed spontaneous cough and/or sneeze-like reflexes in different lines of transgenic mice with immotile cilia and rhinosinusitis [7, 8]. These strikingly common observations suggest that rhinosinusitis including postnasal drip may have caused the cough reflex in these mice. We considered that elucidating mechanism of cough in mice with rhinosinusitis might explain the cause of chronic cough.

Sneezing ejects foreign materials from upper airways, as does cough. However, the neural pathway of sneeze is different from that of cough; cough is evoked by excitation of the vagus nerve while sneeze is evoked by excitation of the trigeminal nerves [9]. Although we easily discriminate cough from sneeze in humans, discrimination in mice is often difficult [10]. General methods to distinguish between cough and sneeze in mice have not yet been reported.

We found characteristic airflow patterns observed in cough, sneeze, and the expiration reflex that differentiated these three reflexes among humans and experimental animals. Our classification of the airflow patterns revealed that the notable reflexes in transgenic mice with immotile cilia consisted mainly of cough. Next, we attempted to identify the cause of cough in these mice. Postnasal drip originating from rhinosinusitis without lower airway inflammation was detected in the pharynx of these mice, indicating that mechanical stimulation by the postnasal drip is the cause of cough in mice with immotile cilia. Finally, the cough reflex was reproduced by artificial postnasal drip in wild-type (WT) mice. Therefore, the present study demonstrated that postnasal drip causes cough.

## Methods

### Animals

Adult male tubulin tyrosine ligase-like family member 1 gene knockout (*Ttll1* ^−/−^) mice [7] (on a C57BL/6 background), C57BL/6 mice (8–30 weeks old, 30–40 g), female WT BALB/c mice (8 weeks old) and male Hartley guinea pigs (330–350 g) were used in this study. Mice and guinea pigs were housed under specific pathogen-free conditions with controlled humidity (50±10% humidity) and temperature (24±2°C) in the Institute of Laboratory Animals Graduate School of Medicine, Kyoto University on a 12-h light/dark cycle with free access to food and water. All experimental procedures were approved by Animal Research Committee, Graduate School of Medicine, Kyoto University (approval numbers are MedKyo 10149, MedKyo 11121 and MedKyo 12123) and were performed according to Regulation on Animal Experimentation at Kyoto University. All surgeries were performed under sodium pentobarbital anesthesia, and all efforts were made to minimize suffering. Mice were sacrificed with an overdose of sodium pentobarbital.

### Cough and other respiratory reflex measurements

A mouse or a guinea pig was placed in a transparent whole body plethysmograph (WBP) (PLY 311 for mouse, PLY 330 for guinea pig; EMMS, Hampshire, UK) that allowed free movement. The plethysmograph was provided airflow at 500 ml/min (in mice) or 1500 ml/min (in guinea pigs). Airflow induced by respiration and reflexes, including cough, sneeze, and the expiration reflex, was detected and recorded by a pneumotachograph (EMMS, Hampshire, UK). Sounds were amplified and recorded using a microphone (EMMS, Hampshire, UK). The behavior of animals was recorded by an external camera (EMMS, Hampshire, UK) eDacq software was used to acquire data [11].

### The mouse and guinea-pig model of cough induced by inhalation of capsaicin or citric acid

Male WT C57BL/6 mice (8–12 weeks old) and male Hartley guinea pigs weighing 330 to 350 g were used. A nebulizer (Aeroneb Lab; Aerogen Inc., Galway, Ireland) was used to expose an animal placed in a WBP to capsaicin (50 μM in mice) or citric acid (0.5 M in guinea pigs) for 10 min in order to evoke cough [11, 12].

### The mouse and guinea pig model of sneeze by allergic rhinitis-induced ovalbumin sensitization

Female wilt-type BALB/c mice (8 week old) and male Hartley guinea pigs weighing 330–350 g were used. Mice were sensitized by an intraperitoneal injection of 2 mg aluminum hydroxide hydrate (ALUM; Cosmo Bio Co., Tokyo, Japan) and 10 μg ovalbumin (WAKO, Osaka, Japan) on days 0, 7, and 14. Guinea pigs were sensitized by 100 mg ALUM and 10 μg ovalbumin on day 0. Sneeze was induced by intranasal instillation of ovalbumin (100 μg/10 μl for mice, 150 μg/15 μl for guinea pigs) on days 21–28 [13, 14].

### Cough, sneeze, and expiration reflex in humans

Healthy male nonsmoking volunteers were recruited (31–40 years old, *n* = 3). A single-use anesthesia face mask (Vital Sighs Inc., Totowa, New Jersey) was attached to a spirometer (ChestGraph HI-701; Chest, Tokyo, Japan), and airflow through the nose and mouth was measured in the FVC mode. The acquired data were converted, and the time course of airflow was represented as a graph. Cough and the expiration reflex were evoked by inhalation of capsaicin (Sigma–Aldrich. St. Louis, Missouri) (300 μM for 5 s). Sneeze was evoked by mechanical stimuli that were applied by rubbing the nasal cavity with a tissue paper. This study was conducted according to the principles expressed in the Declaration of Helsinki and was approved by Ethics Committee, Kyoto University Graduate School and Faculty of Medicine (approval No. 1165). According to the approved procedure, we explained about the study using explanation document and obtained oral informed consent from all participants. The committee exempted us from obtaining written consent considering that the candidates in this study were recruited from our study group and we had to mask name of participants in order to free the candidates of mental bias in their decision. Participant consent was not recorded. Instead, each participant was given study number that was blinded to other study members and the number was recorded with the study result by a technician. The consent procedure was approved by the ethics committee.

### Capsaicin cough sensitivity

In *Ttll1* ^−/−^ and WT mice, we measured the numbers of coughs evoked by nebulizer saline or capsaicin (10 and 50 μM) administered for 10 min at 2 h after counting the pretreatment number of coughs. The increased numbers (posttreatment − pretreatment) were compared between the saline and capsaicin groups or between WT and *Ttll1* ^−/−^ mice.

### Pharmacological examination

The pretreatment number of coughs in *Ttll1* ^−/−^ mice was counted for 10 min at 2 h before drug administration. The posttreatment number of coughs was counted 2 h after oral administration of codeine phosphate (10 mg/kg) (Takeda Pharmaceutical Co., Osaka, Japan) and intraperitoneal administration of moguisteine (3, 10, and 30 mg/kg) and 1 h after intraperitoneal administration of HC-030031 (300 mg/kg). Salbutamol (5 mg/ml) (GlaxoSmithKline, Tokyo, Japan) and capsazepine (300 μM) (C191, Sigma-Aldrich, St. Louis, Missouri) were administered using a nebulizer for 10 min. The posttreatment number of coughs was counted 2 h after the administration of salbutamol and just after the administration of capsazepine. A solution of 4% lidocaine (10 μl) (AstraZeneca, Osaka, Japan) was administered to each nostril, and the posttreatment number of cough was counted 10 min after the administration.

### Measurement of airway resistance

For assessment of enhanced pause (Penh), a mouse was placed in a WBP, and indices of Penh for every 10 s were determined by eDacq software (EMMS, Hampshire, UK). The average for 10 min was calculated in each mouse. For assessment of airway resistance, the flexiVent system (SCIREQ, Montreal, Quebec, Canada) was used to measure airway resistance [15, 16]. Mice were anesthetized with an intraperitoneal injection of pentobarbital sodium (70 mg/kg) and tracheotomized; a 20G catheter was inserted. Mice paralyzed with 0.8 mg/kg pancuronium bromide to block spontaneous breathing were mechanically ventilated with a tidal volume of 10 ml/kg at a rate of 150 breaths / min and with a positive end-expiratory pressure of 2 cm H_2_O. Snapshot perturbation was used to measure airway resistance. To assess airway hyper-responsiveness (AHR), the mice were exposed for 10 s to nebulizer saline and subsequently exposed to increasing concentrations of nebulizer methacholine (2.5, 5, 10, and 20 mg/ml). Airway resistance was measured for 2 min in each nebulization.

### Histology

The resected left lung was inflated to 20 cm H_2_O with 10% neutral buffered formalin (Mildform^®^; WAKO, Osaka, Japan). HE, periodic acid–Schiff and elastic van Gieson’s staining were performed on sagittal sections in the middle of the left lung. Skinned heads of mice were fixed with 10% neutral buffered formalin and decalcified. Coronal sections of the head in the middle between the orbit and nose were stained with HE for the assessment of rhinosinusitis. Sagittal sections of the head were prepared for the assessment of postnasal drip and the larynx.

### Treatment of rhinosinusitis in *Ttll1* ^*−/−*^ mice

Tosufloxacin (50 mg/kg/day) (TOYAMA CHEMICAL CO., Tokyo, Japan) was administered for 7 days to *Ttll1* ^−/−^ mice by gavage. On the 8^th^ day, the number of coughs was counted and the mice were sacrificed to assess nasal and paranasal cavities.

### Artificial postnasal drip model of mice

Polyvinyl alcohol (PVAL) (WAKO, Osaka, Japan) was dissolved in saline. 5 μl of a 5% PVAL solution colored with Evans blue was intranasally administered to unanaesthetised mice. Just after the administration, the mice were sacrificed. The lower jaw with the larynx was dissected to examine the adhesion of the PVAL solution in the upper airway. The larynx was frozen in liquid nitrogen, and sagittal sections of the larynx were prepared for microscopic analysis. A 5% PVAL solution was administered to evoke cough in unanaesthetised and unrestrained WT mice. Cough was assessed by WBP.

### Physical blockade of nasal airway to dam postnasal drip

Euthanised mice were tracheotomized. A thin catheter (24G SURFLO; TERUMO, Tokyo, Japan) was inserted into the tracheotomy orifice to the nasal airway through the larynx, and 5 μl of a contrast material (Iopamiron Injection, iopamidol 755.2 mg/ml) (Bayer, Osaka, Japan) was injected. The LaTheta (LCT-100M) experimental animal computed tomographic system (Aloka, Tokyo, Japan) was used to scan the mice for assessing the position of the contrast material. Following this, *Ttll1* ^−/−^ mice were anesthetized and tracheotomized. A 5-μl aliquot of cyanoacrylate glue (Aron Alpha^®^; Toagosei, Tokyo, Japan) was administered to these mice to dam postnasal drip in the same manner as that described above. As the mice emerged from the anesthetization, we closed the tracheotomy orifice and counted the number of coughs for 10 min. After the experiment, we sacrificed the mice and pathologically examined the blockade of Aron Alpha^®^.

### Bronchoalveolar lavage

The lung was cannulated through the trachea and washed five times with 1 ml of saline. The bronchoalveolar lavage (BAL) fluid was centrifuged, and the cells were stained with Hemacolor^®^ (Merck KGaA, Darmstadt, Germany). A total of 400 cells (neutrophils, eosinophils, macrophages, and lymphocytes) were counted, and the relative numbers of different types of leucocytes were determined.

### Immunohistological staining

The extirpated left lung was inflated to 20 cm H_2_O with OCT compound (Sakura Finetek Japan, Tokyo, Japan) and frozen in isopentane cooled in liquid nitrogen. Sections of 10 μm were fixed with cold acetone and blocked with Protein Block Serum-Free^®^ (Dako, Glostrup, Denmark). They were then incubated with the first antibodies: rabbit antimyeloperoxidase antibodies (1:200, RB-373; Thermo Fisher Scientific, Waltham, Massachusetts) for neutrophil staining, rat antimouse Mac-3 antibodies (1:500; BioLegend, San Diego, California) for macrophage staining, rabbit anti-CD3 polyclonal antibodies (1:100; ab5690, Abcam, Cambridgeshire, UK) and rat anti-CD19 monoclonal antibodies (1:500; ab25232, Abcam, Cambridgeshire, UK) for lymphocyte staining. The EnVision^®^ + system–HRP labeled polymer antirabbit (K4003; DAKO, Glostrup, Denmark) and polyclonal rabbit antirat immunoglobulins (P0450; DAKO, Glostrup, Denmark) were used as the second antibodies. Diaminobenzidine (DAKO, Glostrup, Denmark) was used to visualize the sections, and haematoxylin was used for counterstaining.

### Hydroxyproline (HYP) assay

The right lower lung was homogenised. The lung homogenate was hydrolysed in hydrochloric acid overnight. Chloramines-T was used to oxidise free HYP. The addition of Ehrlich’s reagent resulted in the formation of a chromophore, and the concentration of HYP was measured by the absorbance at 550 nm [17]. Some lung homogenates were used for the protein assay (DC Protein Assay; BioRad, USA). To remove the influence of lung size, we calculated the ratio of HYP to total protein as the HYP index.

### Measurement of smooth muscle area

Three airways with longest diameters from 200 to 400 μm were chosen in each mouse in a random manner, and Image J software was used to measure the smooth muscle layer area and basement membrane length. To remove the influence of airway size, the ratio of smooth muscle layer area to basement membrane length was calculated as the smooth muscle index. The averages of smooth muscle indices were calculated in each mouse.

### The assessment of nasal mucociliary clearance

Mice were anaesthetised, and 2 μl of the contrast material was administered intranasally. The clearance of the contrast material generated by mucociliary transport was measured. The nasal cavity 5 mm from the tip of the nose was scanned by computed tomography (CT) at pretreatment and 30 min and 150 min later, and ImageJ software was used to measure the area of the nasal cavity occupied by the contrast agent. The percent changes in the area of the contrast material were calculated [(area at 30 min −area at 150 min)/area at 30 min]. The mice were kept anaesthetised with an adequate pentobarbital sodium injection so that they did not eject the contrast material because of expiratory reflexes.

Following this, the clearance of the azo dye, Evans blue, administered to the nasal airway was measured. A 10 μl aliquot of Evans blue solution (1 mg/ml) was intranasally administered to anaesthetised mice. The mice were sacrificed 150 min after administration. The nasal cavity was washed with 1 ml formamide, and the stomach was incubated with formamide overnight to collect Evans blue. The concentration of Evans blue in formamide was measured by the absorbance at 620 nm.

### Statistics

Data are expressed as mean ± standard error of mean (S.E.M). Two-tailed Student’s unpaired t-tests, where appropriate, were used to examine inflammatory cells in BAL fluid, indexes of airway resistance, remodeling and nasal clearance. The Mann–Whitney U test was used to analyze the numbers of coughs for comparisons of *Ttll1* ^−/−^ mice with WT mice, and the Wilcoxon signed-rank test was used for paired data obtained within groups before and after treatment. The level of statistical significant was set to *P* < 0.05. Statistical analyses were performed using SPSS (version 20.0, SPSS Inc., Chicago, Illinois) software was used to perform statistical analyses.

## Results

### Cough is distinguished from sneeze and the expiration reflex by airflow pattern

**Fig 1:**
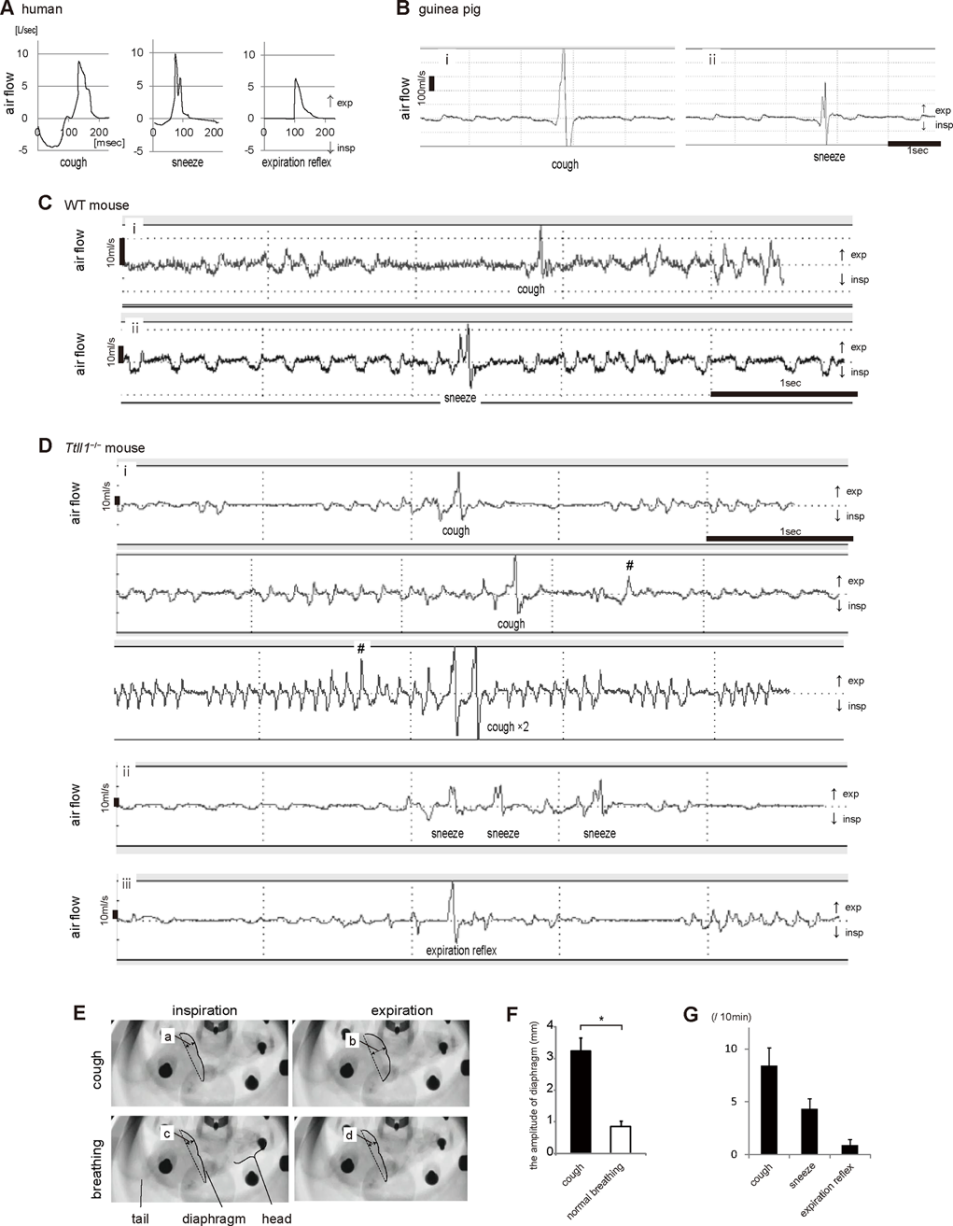
Respiratory reflexes in humans, guinea pigs, wild-type (WT) mice, and *Ttll1* ^*−/−*^ mice. (A−D): Charts exhibiting airflow patterns of respiratory reflexes. Expiratory flow is indicated by the plus sign (upward) and inspiratory flow is indicated by the minus sign (downward). Airflow patterns of human reflexes (A). Airflow through the nose and mouth induced by cough, sneeze, and the expiration reflex was recorded using a spirometer (n = 3). Cough and the expiration reflex were evoked by inhaled capsaicin. Sneeze was evoked by mechanical stimuli applied by rubbing the nasal cavity with a tapered tissue paper. Airflow patterns of reflexes in guinea pigs (B) and WT mice (C; see S1–S4 Videos). Airflows of cough and sneeze were analyzed using a whole body plethysmograph (WBP). Cough was evoked by inhaled citric acid in guinea pigs and capsaicin in mice. Sneeze was induced by intranasal instillation of ovalbumin in sensitized animals. In (A–C), cough and sneeze showed one-peak and two-peak expiration patterns with preceding inspiration, respectively. Airflow patterns of reflexes in *Ttll1* ^−/−^ mice (D; see S5–S7 Videos). These reflexes were analyzed by WBP and classified into three patterns: (i) one-peak expiration with preceding inspiration, (ii) two-peak expiration with preceding inspiration, and (iii) one-peak expiration without preceding inspiration. Patterns (i) and (ii) corresponded to cough and sneeze patterns, respectively. #: expiration during eupneic breathing, which was not accompanied by characteristic sound and motion. (E) Representative photos recorded by videofluoroscopy. The *Ttll1* ^−/−^ mice were placed in a WBP device. Inspiration and expiration phases in cough and normal breathing are shown. Solid lines indicate the diaphragm of the *Ttll1* ^−/−^ mice (see S8 Video). (F) The bar graph shows the calculated amplitude of the diaphragm while coughing [= b–a in (E)] and normal breathing [= d–c in (E)]. The maximal distance [two-headed arrow in (E)] between the dotted line connecting the costophrenic angles and the diaphragm [solid line in (E)] as measured during inspiration and expiration while coughing and normal breathing. Diaphragm motion in the *Ttll1* ^−/−^ mice was larger during coughing than during normal breathing (n = 3; mean ± SEM; *P = 0.006 by two-tailed Student's t-test). (G) Number of respiratory reflexes of the *Ttll1* ^−/−^ mice in ten minutes (mean ± SEM, n = 10).

Respiratory reflexes evoked by stimuli to airways include cough, sneeze, and the expiration reflex. The expiration reflex, mainly evoked by stimuli to the larynx, is usually included as part of cough in clinical research but is discriminated from cough by the absence of preceding inspiration in animal research [18]. Guinea pigs have been traditionally used for the pharmacological or pathophysiological study of cough [3, 19]. Using animal models of cough, it has been reported that trained observers differentiate between cough and sneeze in guinea pigs by assessing the motion and sound of both reflexes [3, 19]. However, an apparent difference in airflow patterns between cough and sneeze has not been documented. In humans, cough is subjectively and empirically distinguished from sneeze by observation of the motion and sound of both reflexes. A clear distinction between cough and sneeze by assessment of the airflow pattern has not been made. Therefore, we investigated differences among airflow patterns of cough, sneeze, and the expiration reflex in humans and experimental animals. First, we used a spirometer to measure airflow through the nose and mouth during respiratory reflexes; cough and the expiration reflex were evoked by inhaled capsaicin while sneeze was evoked by rubbing the nasal cavity. In humans, cough exhibited a one-peak pattern of expiration while sneeze exhibited a two-peak pattern (Fig 1A). The expiration reflex exhibited a one-peak pattern of expiration without preceding inspiration (Fig 1A). Next, we developed guinea pig (Fig 1B i and S1 Video) and mouse (Fig 1C i and S2 Video) models of cough, with cough evoked by inhalation of capsaicin or citric acid. We also prepared guinea pig (Fig 1B ii and S3 Video) and mouse (Fig 1C ii and S4 Video) models of sneeze, with allergic rhinitis induced by ovalbumin sensitization. We used a WBP to analyze the airflow patterns of cough and sneeze. In guinea pigs (Fig 1B) and mice (Fig 1C), cough exhibited one peak and sneeze exhibited two peaks in expiratory airflow patterns, similar to the findings obtained from humans (Fig 1A). Therefore, we found that cough exhibited a one-peak pattern and sneeze exhibited a two-peak pattern in expiratory airflow.

Recently, we reported that *Ttll1* ^−/−^ mice had dysfunctional cilia that resulted in impaired mucociliary clearance and that the mice displayed spontaneous cough- and/or sneeze-like reflexes [7]. Kunimoto et al. also reported similar reflexes in another transgenic mouse with immotile cilia [8]. Because both these mice were affected by rhinosinusitis, we thought that the same mechanism may underlie cough- and/or sneeze-like reflexes. In these reports, the reflexes of these mice were considered to be cough and/or sneeze on the basis of differences in sounds; however, this method was subjective and the airflows of the reflexes had not been assessed [7, 8]. Therefore, whether the phenomena of expiration in *Ttll1* ^−/−^ mice were cough and/or sneeze remained unclear. Using WBP, analysis of the phenomena with characteristic sounds in conscious and unrestrained *Ttll1* ^−/−^ mice revealed three airflow patterns: one-peak expiration with preceding inspiration, pattern 1 (Fig 1D i and S5 Video); two-peak expiration with preceding inspiration, pattern 2 (Fig 1D ii and S6 Video), and one-peak expiration without preceding inspiration, pattern 3 (Fig 1D iii and S7 Video). On the basis of cough and sneeze airflow patterns observed in human and animal models, we considered patterns 1 and 2 to be cough and sneeze, respectively. Pattern 3 was considered to be an expiration reflex according to the absence of preceding inspiration that represents continuous airflow changing without a zero flow plateau between inspiration and expiration.

Up and down movement of the diaphragm induces inspiratory and expiratory airflows in mammals. In humans, motion of the diaphragm during cough, recorded using a videofluoroscope, is more extensive than that during tidal breathing [20]. To confirm that coughing in *Ttll1* ^−/−^ mice was distinct from tidal breathing, we placed the mice in a WBP device and used a videofluoroscope to record the motion of the diaphragm (S8 Video). The motion of the diaphragm during coughing (3.23 ± 0.42 mm) was four times that during tidal breathing (0.85 ± 0.17 mm), and the diaphragm rose particularly during the expiratory phase of cough (Fig 1E and 1F, and S8 Video). This observation verified that this reflex was not normal breathing but was compatible with the cough reflex.

Collectively, these data confirmed that *Ttll1* ^−/−^ mice spontaneously produced cough, sneeze, and the expiration reflex without any artificial stimuli. In *Ttll1* ^−/−^ mice, the number of coughs per 10 min accounted for the majority of reflexes (Fig 1G). These findings indicated that *Ttll1* ^−/−^ mice can be used as an animal model that exhibits spontaneous cough without any artificial stimuli. We considered that further investigation of *Ttll1* ^−/−^ mice may contribute to the understanding of cough mechanisms.

### Cough sensitivity of *Ttll1* ^*−/−*^ mice was increased

**Fig 2:**
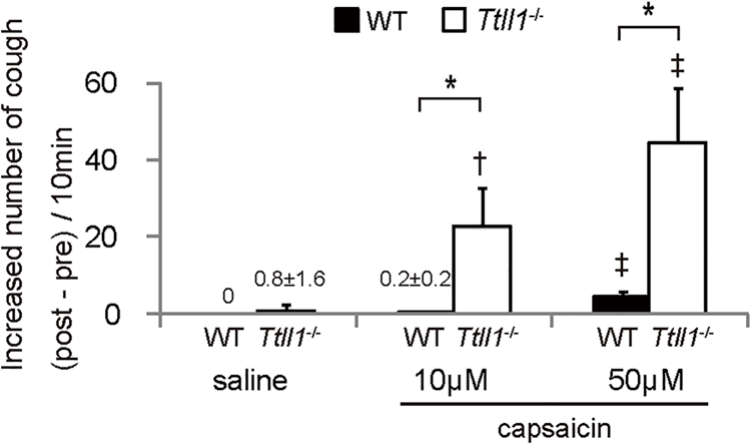
Increased cough sensitivity in *Ttll1* ^−/−^ mice. Wild-type (WT) and *Ttll1* ^−/−^ mice were nebulized with saline and capsaicin (10 and 50 μM). A graph displaying the increased number of coughs (post-treatment–pretreatment). Even low doses (10 μM) of capsaicin increased the number of coughs in the *Ttll1* ^−/−^ mice but not in the WT mice. High doses (50 μM) of capsaicin increased the number of coughs in the WT and *Ttll1* ^−/−^ mice. (n = 5 mice per group; mean ± SEM; *P < 0.05, the increased numbers of coughs were compared between the WT and *Ttll1* ^−/−^ mice; †P < 0.05 and ‡P < 0.01, the increased numbers of coughs in comparison with those induced by saline; Mann–Whitney U-test).

Increased cough sensitivity is one of the factors that contribute to chronic cough [21]. Therefore, we investigated cough sensitivity to capsaicin in *Ttll1* ^−/−^ mice. Cough sensitivity can be assessed by the cough challenge test [3]. Results are represented as C2 and C5, which are the minimum concentrations of tussive agents such as capsaicin and citric acid that cause two or more and five or more coughs, respectively. However, it was difficult to measure the concentrations in *Ttll1* ^−/−^ mice because they were spontaneously coughing without artificial stimulation. Therefore, we provided three preparations (saline only, low dose, and high dose) of capsaicin and assessed evoked cough. In WT mice, a high concentration of capsaicin (50 μM) evoked cough while a low concentration of capsaicin (10 μM) did not (Fig 2). In contrast, in *Ttll1* ^−/−^ mice, even a low concentration of capsaicin significantly increased the number of coughs, as did a high concentration of capsaicin (Fig 2). Furthermore, for each concentration of capsaicin, the number of coughs increased from baseline was markedly higher in the *Ttll1* ^−/−^ mice than in the WT mice (Fig 2). Increased cough response to a lower concentration of capsaicin suggests that cough sensitivity to capsaicin was increased and that hypersensitivity may contribute to chronic cough in *Ttll1* ^−/−^ mice.

### Postnasal drip without inflammation of the lower airways was detected in *Ttll1* ^*−/−*^ mice

**Fig 3:**
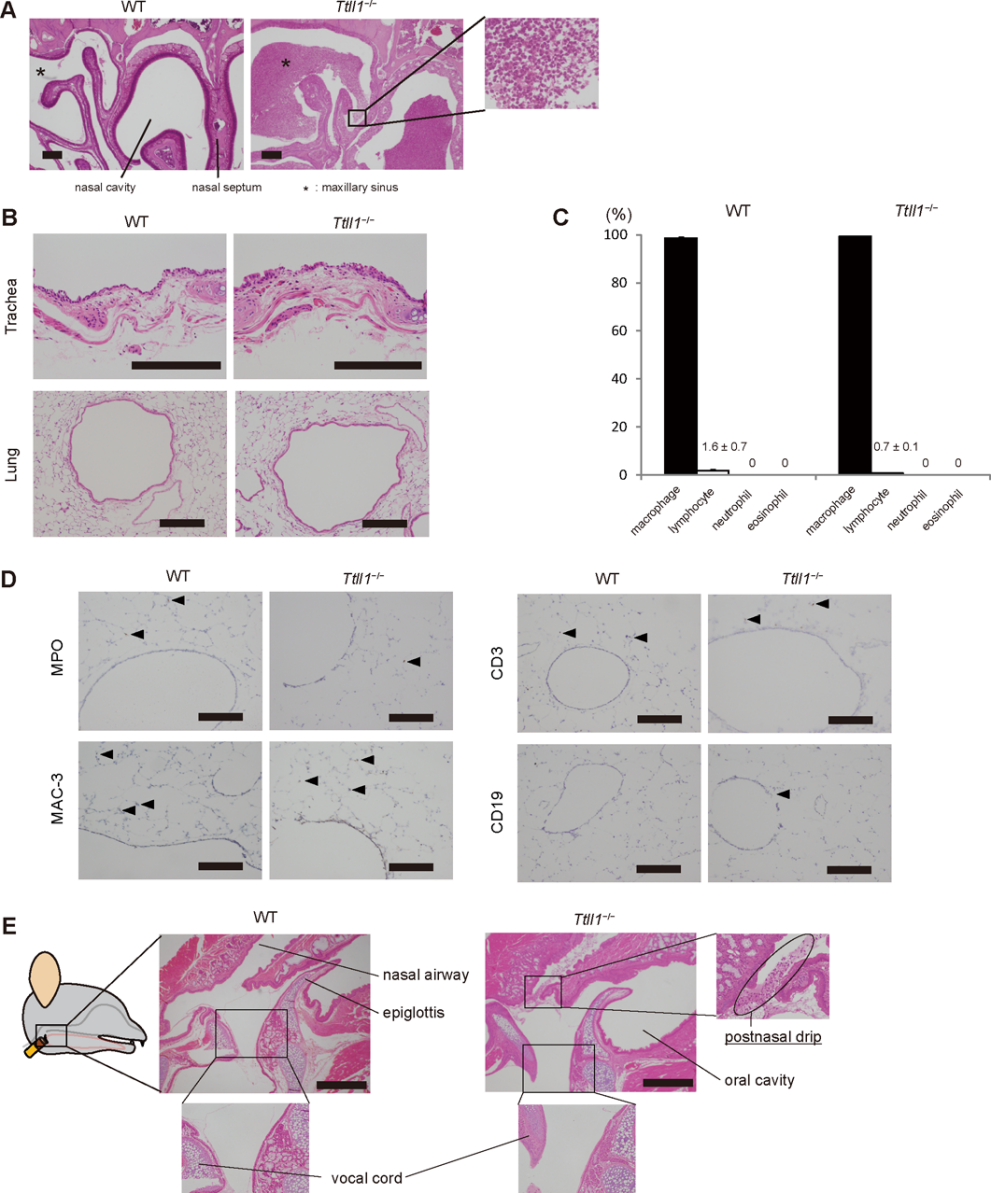
Postnasal drip detected in the pharynx of *Ttll1* ^*−/−*^ mice without lower airway inflammation. (A) Coronal sections of the nasal cavity and paranasal sinus stained with hematoxylin and eosin (HE). Accumulation of mucus and neutrophils were found in the nasal cavity and sinus of the *Ttll1* ^−/−^ mice. Scale bar, 200 μm. (B) Trachea and lung sections stained with HE. Inflammation findings were not observed in the tracheae and lungs of the *Ttll1* ^−/−^ mice. Scale bar, 200 μm. (C) Differential cell counts in bronchoalveolar lavage fluid obtained from the wild-type (WT) and *Ttll1* ^−/−^ mice. There were no significant differences between the WT and *Ttll1* ^−/−^ mice (mean ± SEM, n = 4 mice per group, two-paired Student's t test). (D) Lung sections stained for myeloperoxidase (MPO), Mac-3, CD3, and CD19. A few inflammatory cells (arrows) are detected in the peribronchial region. There were no significant differences between the WT and *Ttll1* ^−/−^ mice. Scale bar, 200 μm. (E) Sagittal sections of the upper airway stained with HE. Postnasal drip characterized by accumulation of mucus and neutrophils was found in the pharyngeal wall. There was no inflammation in the larynx of the *Ttll1* ^−/−^ mice. Scale bar, 500 μm.

As previously reported [7], *Ttll1* ^−/−^ mice were affected with rhinosinusitis (Fig 3A). Because infection of the respiratory tract is a common cause of cough, we histopathologically examined the upper and lower airways of *Ttll1* ^−/−^ mice in detail to determine the causes of cough and sneeze. The trachea and lung sections were stained with hematoxylin and eosin. In contrast to a marked accumulation of mucus and neutrophils in the nasal cavity, we found no accumulation of mucus or findings of inflammation in the trachea or lungs of *Ttll1* ^−/−^ mice. There was no difference in these findings between WT and *Ttll1* ^−/−^ mice (Fig 3B). To confirm that there was no inflammation in the lungs, we evaluated cell differentials in BAL fluid and immunohistologically assessed neutrophils (myeloperoxidase-positive cells), macrophages (Mac-3–positive cells), and lymphocytes (CD3-positive or CD19-positive cells) in the lung. We found no apparent differences in differential cell counts in the BAL fluid or in immunohistological examination findings between the WT and *Ttll1* ^−/−^ mice (Fig 3C and 3D). Therefore, inflammation of the lower airways in *Ttll1* ^−/−^ mice was not observed, and we concluded that inflammation of the lower airways was not the cause of cough in *Ttll1* ^−/−^ mice.

Next, we analyzed sagittal sections of the upper airways, including the pharynx and larynx. Because the larynx has vagal afferent nerves that regulate cough, laryngeal inflammation may be the cause of cough. However, we found no indication of inflammation in the larynx of the *Ttll1* ^−/−^ mice (Fig 3E). One of the mechanisms of cough associated with rhinosinusitis is postnasal drip stimulation of cough receptors located in the hypopharynx and larynx [22]. We found postnasal drip, which consists of mucus and neutrophils, in the pharynx in five of 10 dissected *Ttll1* ^−/−^ mice (Fig 3E). Pathological examination revealed that postnasal drip without laryngeal or lower airway inflammation was the cause of cough in the *Ttll1* ^−/−^ mice.

**Fig 4:**
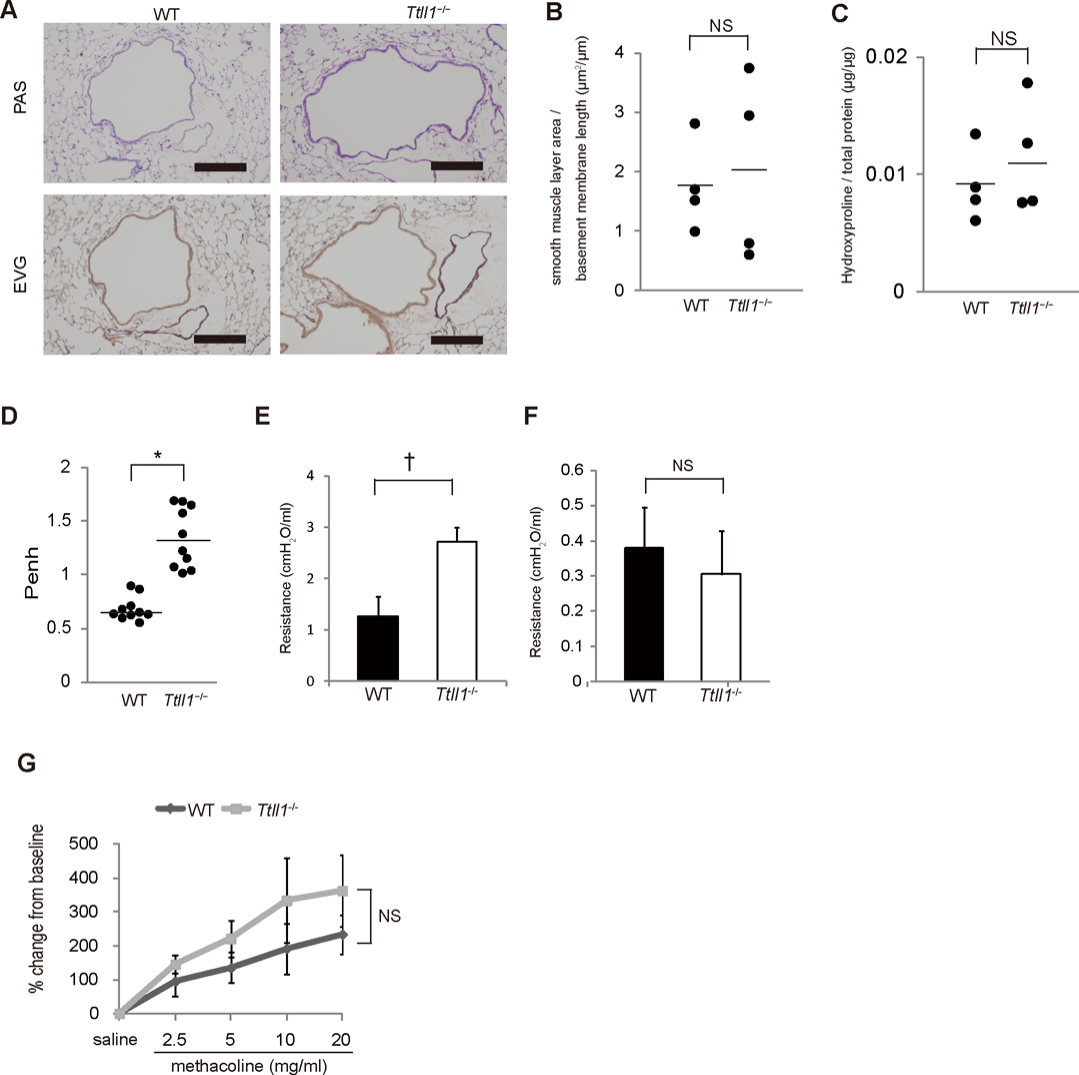
Increased upper airway resistance without airway remodeling in *Ttll1* ^−/−^ mice. (A) Lung sections stained with periodic acid-Schiff (PAS) and elastic van Gieson's (EVG) stains. There were no apparent differences between the wild-type (WT) and *Ttll1* ^−/−^ mice (n = 4 per group). Scale bar, 200 μm. (B) Measurement of smooth muscle area. To adjust airway size, the area of the smooth muscle layer was divided by the length of the basement membrane (bars indicate mean values, n = 4 per group, two-tailed Student's t test; NS = not significant). (C) Measurements of hydroxyproline in lung homogenates. Right lower lung homogenates used for the hydroxyproline assay (bars indicate mean values, n = 4 per group, two-tailed Student's t test) (D). The baseline enhanced pause (Penh) indices of mice (bars indicate mean values, n = 10 mice per group, * P < 0.0001 by two-tailed Student's t-test). (E and F) The flexiVent system was used to measure upper (E) and lower (F) airway resistance in the anesthetized and intubated mice. The upper airway resistance was increased, but lower airway resistance was not increased in the *Ttll1* ^−/−^ mice compared with that in the WT mice (mean ± SEM, n = 4 mice per group, † P < 0.05 by two-tailed Student's t-test). (G) Assessment of airway hyperresponsiveness (AHP). The mice were exposed to nebulized saline and methacholine (2.5, 5, 10, 20 mg/ml), and airway resistance was measured in each nebulization. There was no difference in AHP to methacholine (mean ± SEM, n = 4 mice per group, two-tailed Student's t-test; NS = not significant).

We reported that airway remodeling such as subbasement membrane thickening, goblet cell hyperplasia, and airway smooth muscle hypertrophy was found in patients with chronic cough [23, 24]. Recently, Grainge et al. reported that mechanical stimuli without inflammation induced subepithelial collagen band thickening and goblet cell hyperplasia in patients with asthma [25]. It appears from these reports that mechanical stress induced by cough may lead to remodeling of the airways. Therefore, we considered that continuous mechanical stress associated with the cough in the *Ttll1* ^−/−^ mice may have led to the development of airway remodeling. We examined goblet cells, subbasement membrane, and smooth muscle cells in the bronchus and the amount of hydroxyproline in the lungs of the *Ttll1* ^−/−^ mice to assess airway remodeling (Fig 4A−4C), but contrary to our expectation, there were no findings of airway remodeling.

We analyzed airway resistance and AHR to investigate the physiological characteristics of *Ttll1* ^−/−^ mice. A dimensionless parameter that correlates with airway resistance is Penh [26]. Penh values were significantly higher in the *Ttll1* ^−/−^ mice than in the WT mice (Fig 4D). Generally, an increased Penh value represents increased lower airway resistance; however, if mice are affected with upper airway diseases, the Penh value increases without an increase in lower airway resistance [27]. Therefore, we used the FlexiVent system to measure upper and lower airway resistance in the *Ttll1* ^−/−^ mice [15, 16]. Compared with the WT mice, upper airway resistance was increased, while lower airway resistance was not, in the *Ttll1* ^−/−^ mice (Fig 4E and 4F). In anatomical analyses, we did not find any significant differences in airways of *Ttll1* ^−/−^ mice except for rhinosinusitis (Fig 3). Although mechanical differences in the pharynx or larynx of *Ttll1* ^−/−^ mice remain, upper airway obstruction originating from rhinosinusitis and postnasal drip appears to be one of the causes of the increased Penh values in the *Ttll1* ^−/−^ mice.

AHR occurs when lower doses of agents such as methacholine and histamine induce bronchoconstriction in affected subjects compared with normal subjects. AHR is a major feature of patients with cough variant asthma, which is one of the causes of chronic cough [28]. We assessed whether AHR is associated with cough in *Ttll1* ^−/−^ mice. There was no difference in airway responsiveness to methacholine between the WT and *Ttll1* ^−/−^ mice (Fig 4G). This result indicated that AHR, a classic physiological finding in asthma, was not the cause of cough in the *Ttll1* ^−/−^ mice. Collectively, absence of lower airway inflammation and AHR strongly suggested that cough was not caused by lower airway diseases but by rhinosinusitis.

### Cough responses to antitussives in *Ttll1* ^*−/−*^ mice

**Fig 5:**
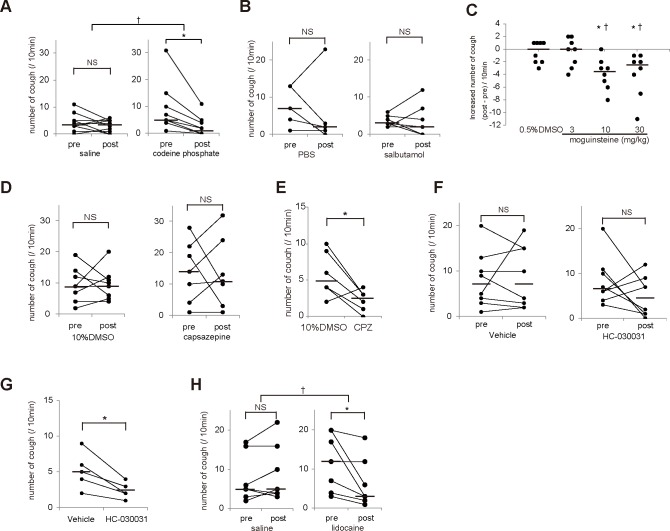
Effects of antitussive drugs on cough in *Ttll1* ^−/−^ mice. (A, B, D, F, H) Graphs displaying the pre- and post-treatment number of coughs. (C) Graph displaying the dose–response relationship of the increased number of coughs in *Ttll1* ^−/−^ mice. (E, G) Graph displaying the number of coughs evoked by inhaled capsaicin in wild-type (WT) mice treated with control and capsazepine (CPZ) or HC-030031. (A) Codeine phosphate (10 mg/kg) or saline was administered by gavage. Codeine phosphate significantly decreased cough in the *Ttll1* ^−/−^ mice. (B) The *Ttll1* ^−/−^ mice were nebulized with salbutamol (5 mg/ml) or phosphate-buffered saline (PBS). Salbutamol did not decrease cough in the *Ttll1* ^−/−^ mice. (C) Moguisteine (3, 10, and 30 mg/kg) or control [0.5% dimethylsulfoxide (DMSO)] was intraperitoneally administered. Administration of 10 and 30 mg/kg moguisteine significantly inhibited cough in the *Ttll1* ^−/−^ mice. (D) The *Ttll1* ^−/−^ mice were nebulized with CPZ (300 μM) or control (10% DMSO). CPZ did not decrease cough in the *Ttll1* ^−/−^ mice. (E) After treatment with nebulized CPZ (300 μM) or control (10% DMSO), the WT mice were nebulized with capsaicin to evoke cough. Nebulization with 300 μM CPZ was sufficient to inhibit cough evoked by capsaicin in the WT mice. (F) Vehicle (0.5% methyl cellulose in sterile saline) or HC-030031 (300 mg/kg) was administered intraperitoneally to the *Ttll1* ^−/−^ mice. HC-030031 did not decrease cough in the *Ttll1* ^−/−^ mice. (G) After administration of HC-030031 (300 mg/kg) or vehicle, the WT mice were nebulized with acrolein (10 mM) to evoke cough. Administration of HC-030031 was sufficient to inhibit cough evoked by acrolein in the WT mice. (H) Lidocaine (4%) or saline was administered to each nostril. Lidocaine decreased cough in the *Ttll1* ^−/−^ mice [bars indicate median values; n = 5–10 mice in each group; *P < 0.05, number of coughs compared between pre- and post-treatment (in A−D, F, H) or between control and treated groups (in E and G) using Wilcoxon signed-rank test; †P < 0.05, increased number (post-treatment–pretreatment) compared with that in the control group using Mann–Whitney U-test; NS = not significant].

We pharmacologically investigated the cause of cough in *Ttll1* ^−/−^ mice. Opioid receptors mediate the effects of codeine phosphate, which acts centrally and is one of the most common antitussives. Cough was significantly decreased in the *Ttll1* ^−/−^ mice treated with orally administered codeine phosphate (10 mg/kg) compared with that in the vehicle-treated group (Fig 5A). This result was compatible with the finding that the reflex observed in the *Ttll1* ^−/−^ mice was cough because it was reported that codeine did not suppress sneeze and the expiration reflex [29, 30]. Bronchodilators are effective for inhibiting cough in patients affected by cough variant asthma [31]. Inhalation of salbutamol did not inhibit cough in the *Ttll1* ^−/−^ mice (Fig 5B). Therefore, we thought that the pathogenesis of cough in the *Ttll1* ^−/−^ mice was different from that in patients with cough variant asthma or classical asthma, which is compatible with the absence of AHR to methacholine (Fig 4G).

Tussive stimuli are mediated in the brainstem through Aδ-fibers and/or c-fibers, both of which comprise vagal afferent nerves [32–35]. C-fibers are sensitive to chemical stimuli such as capsaicin, bradykinin, and acrolein, which is present in air pollution, whereas Aδ-fibers are activated by mechanical stimuli caused by bronchospasm, mucus accumulation, vasodilatation, and edema [33, 36–38]. Moguisteine inhibited cough induced by capsaicin, but did not affect the cardiovascular and respiratory responses to capsaicin [39]. This finding suggests that moguisteine does not inhibit c-fiber activation induced by the transient receptor potential vanilloid 1 (TRPV1) [39]. Cough was also inhibited by capsazepine, an antagonist of TRPV1, or HC-030031, an antagonist of the transient receptor potential cation channel, subfamily A, member 1, located in the terminal of c-fibers [36, 40]. Intraperitoneal administration of moguisteine inhibited cough in *Ttll1* ^−/−^ mice (Fig 5C), whereas cough was not inhibited by administration of capsazepine or HC-030031 (Fig 5D and 5F) even at the dose that sufficiently inhibited cough evoked by the inhalation of capsaicin or acrolein, respectively, in the WT mice (Fig 5E and 5G). These results suggest that cough in the *Ttll1* ^−/−^ mice may have been evoked by the activation of Aδ-fibers rather than c-fibers.

Local anesthetics such as lidocaine, bupivacaine, and mexiletine, block voltage-gated sodium channels and inhibit the generation and transmission of action potential on peripheral nerves [41]. We intranasally administered lidocaine to the *Ttll1* ^−/−^ mice and found that it inhibited cough (Fig 5H). Lidocaine administered intranasally acted on peripheral sites such as the nasal airway, pharynx, larynx, and trachea. Therefore, the cough reflex was thought to be evoked by stimuli in these peripheral sites. On the basis of all these results, we think that peripheral mechanical stimuli associated with postnasal drip evoke cough through RARs in *Ttll1* ^−/−^ mice.

### Cough is evoked by mechanical stimuli associated with postnasal drip

**Fig 6:**
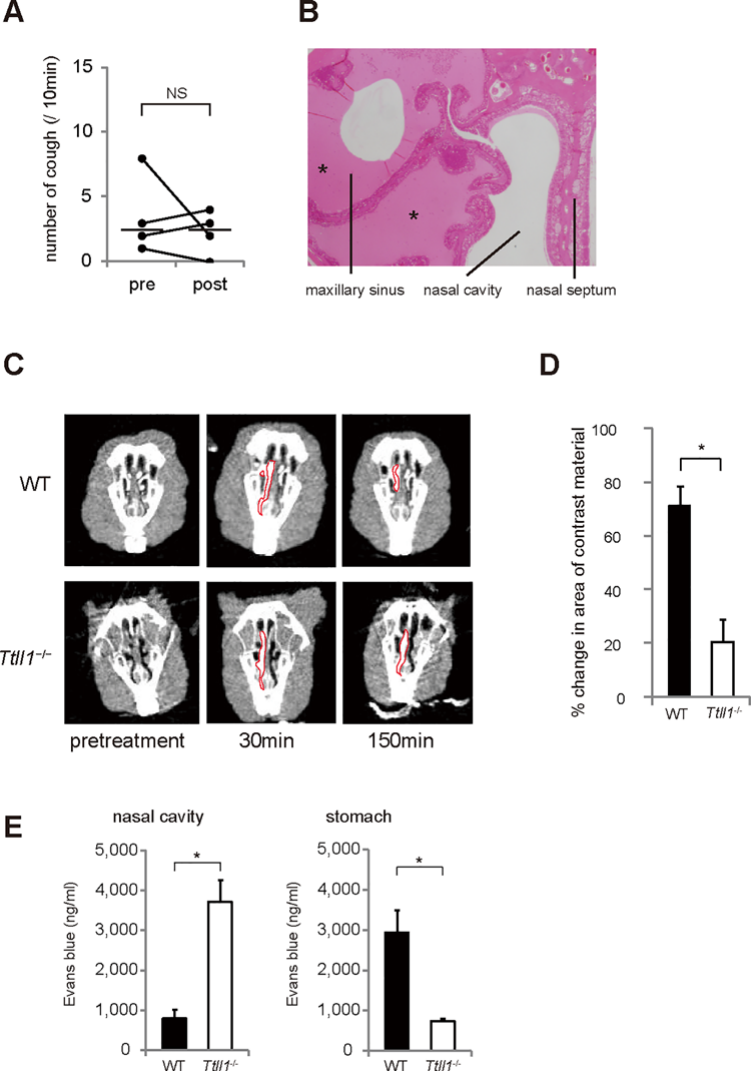
Nasal mucociliary clearance was decreased in *Ttll1* ^−/−^ mice. (A) Tosufloxacin (50 mg/kg) was administered to *Ttll1* ^−/−^ mice with rhinosinusitis to inhibit cough for 7 days. Treatment with tosufloxacin did not decrease cough (bars indicate median values, n = 4 per group; NS = not significant). (B) Nasal sections (coronal) of the *Ttll1* ^−/−^ mice treated with tosufloxacin. Neutrophils were decreased in the mucus. However, mucus accumulation persisted (*) (n = 4). (C) Representative computed tomography scans of the coronal nasal cavity. The area occupied by contrast material is bordered with a red line. (D) We calculated the percent changes in the area of contrast material [(area at 30 min − area at 150 min)/area at 30 min] to assess clearance of contrast material (mean ± SEM, n = 4, *P < 0.005 by two-tailed Student's t test). (E) Concentration of Evans blue in the nasal cavity and stomach 90 min after administration. In the *Ttll1* ^−/−^ mice, a larger amount of Evans blue persisted in the nasal cavity and a lesser amount was swallowed compared with that in the wild-type (WT) mice (mean ± SEM, n = 5, *P < 0.005 by two-tailed Student's t test).

Because rhinosinusitis linked to postnasal drip appeared to be the cause of cough in the *Ttll1* ^−/−^ mice, we hypothesized that improvement in nasal inflammation can decrease cough in *Ttll1* ^−/−^ mice. However, treatment of rhinosinusitis with tosufloxacin, a fluoroquinolone antibiotic, did not decrease cough in the *Ttll1* ^−/−^ mice (Fig 6A). A reduction in neutrophils was observed in nasal discharge fluid, whereas accumulation of mucus persisted in nasal cavities and sinuses (Fig 6B). Because impaired mucociliary clearance seemed to be the cause of the accumulated mucus, we examined whether ciliary dyskinesia in the nasal epithelium of the *Ttll1* ^−/−^ mice resulted in impaired nasal mucociliary clearance. Nasal mucociliary clearance of a contrast agent administered to the nasal cavity was decreased in the *Ttll1* ^−/−^ mice as assessed by CT (Fig 6C and 6D). We confirmed that nasal discharge, which runs into the larynx as postnasal drip, was eventually swallowed into the stomach (Fig 6E). These results indicated that the accumulation of mucus because of impaired mucociliary clearance, and not neutrophilic inflammation, evoked cough in the *Ttll1* ^−/−^ mice.

**Fig 7:**
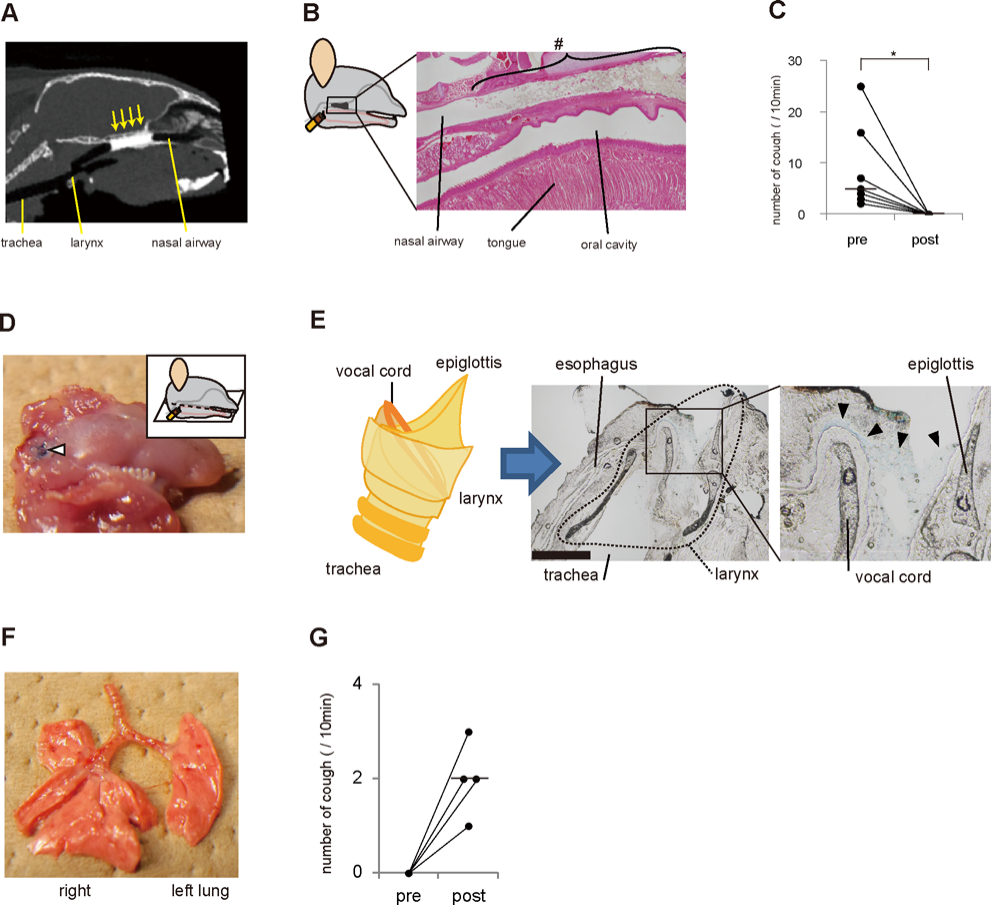
Laryngeal stimuli by postnasal drip-evoked cough in mice. (A) Contrast material in the nasal airway and upper airway was scanned by computed tomography (CT). CT shows optimal nasal airway obstruction with contrast material (arrows). (B) Physical blockade of the nasal airway to dam postnasal drip. Cyanoacrylate glue was placed in the nasal airway of *Ttll1* ^−/−^ mice, as illustrated. Sagittal section of the nasal airway stained with hematoxylin and eosin showing obstruction of the nasal airway with cyanoacrylate glue (#). (C) Graph displaying the pre- and post-treatment number of coughs in the *Ttll1* ^−/−^ mice. Cough in the *Ttll1* ^−/−^ mice was completely inhibited by nasal airway blockade with cyanoacrylate glue (bars: median values, n = 7, *P = 0.02 by Wilcoxon signed-rank test). (D−F) Artificial postnasal drip in the wild-type (WT) mice (n = 5). A blue-colored polyvinyl alcohol (PVAL) solution was intranasally administered to the WT mice to mimic postnasal drip. The photos show the lower jaw (D) and lung (F), and the photomicrographs show sagittal sections of the larynx (E) after administration of the PVAL solution. The PVAL solution (blue) was found in the larynx (white and black arrowheads). Bar, 1 mm. There was no finding of aspiration of the PVAL solution in the trachea and lungs (E and F). (G) Graph showing the pre- and post-treatment number of coughs in the WT mice. A PVAL solution (artificial postnasal drip) was intranasally administered to the WT mice. Cough was evoked by an artificial postnasal drip in the WT mice (n = 4).

Because accumulated mucus was suspected to be the cause of postnasal drip and consequent cough, we attempted to inhibit cough in the *Ttll1* ^−/−^ mice by damming nasal discharge to prevent it from running into the larynx. Administration of 5 μl of a contrast agent by a thin catheter inserted through a tracheotomy orifice obstructed the nasal airway optimally as assessed by CT scans (Fig 7A). Next, we obstructed the nasal airway with the same amount of cyanoacrylate glue to dam the nasal discharge (Fig 7B). Cyanoacrylate glue is commonly used in surgical intervention, and endoscopic application of the material is reportedly effective for closing bronchopleural fistulae [42]. This airway obstruction successfully inhibited cough in the *Ttll1* ^−/−^ mice treated with cyanoacrylate glue (Fig 7C). Because inflammation of the nasal cavity and sinuses was not suppressed by this airway obstruction, we thought that mechanical stimuli due to postnasal drip rather than nasal neutrophilic inflammation evoked cough. To confirm that postnasal drip evoked cough, we generated an artificial postnasal drip model in WT mice and investigated whether postnasal drip evoked cough. We used a PVAL solution to mimic nasal discharge. A PVAL solution is a viscous material contained in ophthalmic artificial tear solutions. When administered intranasally to the WT mice, 5 μl of the blue-colored PVAL solution attached to the larynx (Fig 7D and 7E). The solution was not aspirated, as evidenced by the observation that the blue solution was not detected in the trachea and lungs (Fig 7E and 7F). In the WT mice, intranasal administration of the PVAL solution evoked cough as detected by WBP (Fig 7G). Collectively, these data show that mechanical stimuli caused by postnasal drip itself stimulated the larynx and evoked cough in the mice.

## Discussion

We experimentally demonstrated that cough was evoked by mechanical stimuli due to postnasal drip from rhinosinusitis. We believe that elucidation of the mechanism underlying chronic cough in *Ttll1* ^−/−^ mice may also help us understand pathophysiological mechanisms in patients with rhinosinusitis. We identified the same patterns of airflow in cough, sneeze, and the expiration reflex among humans, guinea pigs, and mice. First, a spirometer was used to record cough, sneeze, and the expiration reflex in humans. Second, animal models of cough evoked by capsaicin or citric acid were investigated. Third, animal models of sneeze caused by allergic rhinitis induced by ovalbumin sensitization were examined. These results revealed that cough exhibited a one-peak pattern of expiration with preceding inspiration, whereas sneeze exhibited a two-peak pattern of expiration with preceding inspiration. The expiration reflex exhibited one-peak expiration without preceding inspiration. Sneeze evoked by stimuli to the nasal mucosa is a protective reflex to eliminate foreign bodies and nasal discharge. While sneezing, closing of the nasopharynx by elevation of the back of the tongue followed by rapid opening of the upper airway during the expiration phase creates strong airway pressure to produce expulsive nasal airflow [43]. We assumed that upper airway closure, which is unique to sneeze and not observed in cough, caused the two-peak pattern of expiration in sneeze. No studies have shown that cough, sneeze, and the expiration reflex have common airflow patterns, regardless of the animal species.

Most investigators have concluded that mice do not cough and the respiratory reflexes that are observed in mice and rats are regarded as expiration reflexes rather than true cough [3]. In 1979, Korpáés et al. reported that mechanical stimulation did not produce a cough reaction in mice or rats [10]. However, in their experiments, mice were anethetized and changes in interpleural pressure following mechanical stimulation were recorded to detect cough [10]. This invasive method involving anethesia might have negatively affected their sensitivity to detect cough in mice. Chemical stimulation with sulfur dioxide also failed to evoke cough in mice [10]. On the other hand, our method utilized WBP to simultaneously record airflow, sound, and behavior without a requirement for mouse restraint or anethesia. This noninvasive examination in a more natural setting may have helped us to detect cough in mice. Indeed, a recently reported study used WBP to detect capsaicin-induced cough in mice [44]. In addition, we confirmed the characteristic motion of the mouse diaphragm during the cough reflex using a videofluoroscope (Fig 1E and 1F; and S8 Video). This result supports the WBP findings indicating that the observed reflexes that were regarded as true cough were not a part of eupneic breathing or expiration reflexes. Thus, the multidirectional and comprehensive observation methods employed in this study confirmed that mice do cough in experimental settings.

In humans, postnasal drip does not always induce cough [45]. Humans can breathe through the mouth when they are affected by rhinosinusitis with postnasal drip and nasal obstruction, but mice generally do not breathe through the mouth. Therefore, humans can protect themselves from increased postnasal drip, whereas postnasal drip in mice with rhinosinusitis tends to run into their larynx because of nasal breathing. Therefore, cough may be evoked by postnasal drip more easily in mice than in humans.

Several mechanisms of cough evoked by rhinosinusitis in humans have been proposed without clear evidence: stimuli of postnasal drip to receptors in the larynx, aspiration of postnasal drip into the lower airway, and increased cough sensitivity in patients with rhinitis [6]. In experiments using mice, intranasal instillation is a common technique for the administration of various agents into lower airways. It was reported that intranasal instillation of 5 μl was not aspirated into the lung and that more than 5 μl was necessary to deliver agents to the lung in lightly anesthetized mice [46]. In our study, because we administered 5 μl of artificial postnasal drip to mice without anesthesia, these mice did not aspirate the agents into their lungs. Furthermore, because the dye inoculated into the nose was not aspirated into the lower airway (Fig 7E and 7F) and because inflammation findings were not detected in the lower airways in our experiments (Fig 3B−3D), it was unlikely that cough in the *Ttll1* ^−/−^ mice was evoked by the aspiration of postnasal drip. In electron microscopic examination of the airway epithelium of mice, intraepithelial nerve endings were not found in the trachea, with the exception of the larynx [47]. This absence of intraepithelial nerve endings may exclude the possibility that cough is evoked by the aspiration of postnasal drip.

Rhinitis or stimulus to the trigeminal nerves serves to increase cough sensitivity [48, 49]. Consistent with a previous report that showed increased cough sensitivity in patients with allergic rhinitis [48], the *Ttll1* ^−/−^ mice affected with rhinosinusitis showed increased cough sensitivity to capsaicin (Fig 2). There was no apparent inflammation in the larynx of the *Ttll1* ^−/−^ mice, suggesting that laryngitis was not involved in the increased cough sensitivity (Fig 3E). Therefore, to decrease cough, treatment of nasal inflammation may be important for normalization of increased cough sensitivity. Blockade of nasal airways to dam postnasal drip completely inhibited cough in the *Ttll1* ^−/−^ mice, whereas the blockade did not improve rhinosinusitis as expected. Although rhinosinusitis is probably involved in increased cough sensitivity, it was unlikely to be the direct trigger of cough. On the other hand, we showed that artificial postnasal drip obviously evoked cough in the WT mice without rhinosinusitis. These results indicate that postnasal drip served as the direct trigger of cough.

Bronchopulmonary Aδ-fibers, including rapidly adapting airway mechanoreceptors and touch-sensitive tracheal Aδ-fibers, and c-fibers have been implicated in cough [33, 50]. In our study, moguisteine decreased cough, but neither the TRPV1 nor TRPA1 antagonists inhibited cough in *Ttll1* ^−/−^ mice. These results suggest that cough induced in *Ttll1* ^−/−^ mice is evoked through the activation of Aδ-fibers rather than c-fibers. The efficacy of moguisteine in suppressing cough evoked by mechanical stimulation via Aδ-fibers has not been reported. To demonstrate that touch-sensitive tracheal Aδ-fibers facilitate cough in *Ttll1* ^−/−^ mice, an experiment such as direct mechanical punctuation in anesthetized mice would be ideal. However, we were unable to perform this type of examination using our method incorporating WBP to detect cough. Instead, we showed that intranasal administration of the PVAL solution evoked cough in WT mice. Taken together, mechanical stimuli caused by postnasal drip evoked cough in *Ttll1* ^−/−^ mice potentially, but not definitively, via Aδ-fibers.

Airway remodeling such as sub-basement membrane thickening, goblet cell hyperplasia, and airway smooth muscle hypertrophy was observed in patients with chronic cough [23, 24]. It is not known whether chronic cough resulted in airway remodeling or whether airway remodeling was the cause of chronic cough in these patients. Mechanical stress to bronchial epithelial cells induced the expression of genes related to airway remodeling, such as transforming growth factor-β and metalloproteinase-9, in airway epithelium in *in vitro* studies [51, 52]. Chronic cough in *Ttll1* ^−/−^ mice seems to cause mechanical stress in the airway and contribute to the development of airway remodeling, but we did not find any findings related to airway remodeling. In this study, we analyzed the airways of mice in the reproductive period. The median duration of cough in a human study on airway remodeling due to nonasthmatic chronic cough was 8 years [24], which was much longer than the median duration of cough in this study. The short duration of cough in our study may be the reason for the absence of airway remodeling in the *Ttll1* ^−/−^ mice.

Primary ciliary dyskinesia is a hereditary disease characterized by immotile cilia. Patients with this condition have been affected by pulmonary diseases from the time they were neonates, and bronchiectasis is frequently observed in adult patients [53]. The *Ttll1* ^−/−^ mice were not affected by these lower airway diseases. One of the reasons for the absence of lower airway diseases may have been that ciliary beating and cilia-generated clearance were partially maintained [7]. In addition, cough may play an important role in protection against infection and accumulation of mucus [54].

In conclusion, we found that cough and sneeze showed one- and two-peak expiration airflow patterns, respectively, in humans, guinea pigs, and mice. The criteria for discrimination between cough and sneeze revealed that the mice with immotile cilia coughed spontaneously because of postnasal drip. Cough was evoked by reproduction of postnasal drip in the WT mice. We have used an experimental mouse model to demonstrate that sensation of mechanical stimulation by postnasal drip in the larynx is transmitted to the central nervous system. Understanding the mechanism of cough by postnasal drip may provide a novel strategy for the treatment of chronic cough in patients with rhinosinusitis.

## Supporting Information

S1 Checklist Completed “The ARRIVE Guidelines Checklist” for reporting animal data in this manuscript. (PDF)

S1 Video Guinea pig model of cough induced by citric acid. A guinea pig was placed in a whole body plethysmograph (WBP). Cough is induced by inhalation of citric acid in a guinea pig. The upper and lower charts exhibit the audio and airflow signals, respectively. In the airflow signal, expiratory flow is indicated by a plus (upward), and inspiratory flow is indicated by a minus (downward). Cough exhibits a one-peak pattern of expiration with preceding inspiration.
(MOV)

S2 Video Mouse model of cough induced by capsaicin. A wild-type (WT) mouse was placed in a WBP. Cough is induced by inhalation of capsaicin in a WT mouse. The direction of airflow is as indicated in S1 Video. Cough exhibits a one-peak pattern of expiration with preceding inspiration.
(MOV)

S3 Video Guinea pig model of sneeze induced by ovalbumin. A guinea pig was placed in a WBP. Sneeze is induced by nasal administration of an ovalbumin solution to a sensitized guinea pig. The direction of airflow is as indicated in S1 Video. Sneeze exhibits a two-peak pattern of expiration with preceding inspiration.
(MOV)

S4 Video Mouse model of sneeze induced by ovalbumin. A WT mouse was placed in a WBP. Sneeze is induced by nasal administration of an ovalbumin solution to a sensitized WT mouse. The direction of airflow is as indicated in S1 Video. Sneeze exhibits a two-peak pattern of expiration with preceding inspiration.
(MOV)

S5 Video Cough of *Ttll1* ^−/−^ mice. A *Ttll1* ^−/−^ mouse was placed in a WBP. Cough is spontaneously observed in *Ttll1* ^−/−^ mice without any stimuli. The direction of airflow is as indicated in S1 Video. Cough exhibits a one-peak pattern of expiration with preceding inspiration.
(MOV)

S6 Video Sneeze of *Ttll1* ^−/−^ mice. A *Ttll1* ^−/−^ mouse was placed in a WBP. Sneeze is spontaneously observed in *Ttll1* ^−/−^ mice without any stimuli. This movie contains three consecutive sneezes. The direction of airflow is as indicated in S1 Video. Sneeze exhibits a two-peak pattern of expiration with preceding inspiration.
(MOV)

S7 Video The expiration reflex of *Ttll1* ^−/−^ mice. A *Ttll1* ^−/−^ mouse was placed in a WBP. The expiration reflex is spontaneously observed in *Ttll1* ^−/−^ mice without any stimuli. The direction of airflow is as indicated in S1 Video. The expiration reflex exhibits a one-peak pattern of expiration without preceding inspiration.
(MOV)

S8 Video The motion of the diaphragm of *Ttll1* ^−/−^ mice. A *Ttll1* ^−/−^ mouse was put into a WBP and the system was placed into a videofluoroscope. Tidal breathing and cough were recorded. This movie contains three consecutive coughs, which were determined by WBP. The motion of the diaphragm is larger during coughing than during tidal breathing. See also Fig 1E and 1F.
(MOV)
